# Nanoradiopharmaceuticals Based on Alpha Emitters: Recent Developments for Medical Applications

**DOI:** 10.3390/pharmaceutics13081123

**Published:** 2021-07-23

**Authors:** Maydelid Trujillo-Nolasco, Enrique Morales-Avila, Pedro Cruz-Nova, Kattesh V. Katti, Blanca Ocampo-García

**Affiliations:** 1 Departamento de Materiales Radiactivos, Instituto Nacional de Investigaciones Nucleares, Carretera México-Toluca S/N, Ocoyoacac 52750, Mexico; mayis421@gmail.com (M.T.-N.); pedro.nova1990@gmail.com (P.C.-N.); 2 Facultad de Química, Universidad Autónoma del Estado de México, Paseo Tollocan S/N, Toluca 50120, Mexico; emoralesav@uaemex.mx; 3 Department of Radiology, Institute of Green Nanotechnology, University of Missouri, Columbia, MO 65212, USA; KattiK@health.missouri.edu

**Keywords:** targeted alpha-particle therapy (TAT), recoil energy, nanoparticles

## Abstract

The application of nanotechnology in nuclear medicine offers attractive therapeutic opportunities for the treatment of various diseases, including cancer. Indeed, nanoparticles-conjugated targeted alpha-particle therapy (TAT) would be ideal for localized cell killing due to high linear energy transfer and short ranges of alpha emitters. New approaches in radiolabeling are necessary because chemical radiolabeling techniques are rendered sub-optimal due to the presence of recoil energy generated by alpha decay, which causes chemical bonds to break. This review attempts to cover, in a concise fashion, various aspects of physics, radiobiology, and production of alpha emitters, as well as highlight the main problems they present, with possible new approaches to mitigate those problems. Special emphasis is placed on the strategies proposed for managing recoil energy. We will also provide an account of the recent studies in vitro and in vivo preclinical investigations of α-particle therapy delivered by various nanosystems from different materials, including inorganic nanoparticles, liposomes, and polymersomes, and some carbon-based systems are also summarized.

## 1. Introduction

Interest in theranostics and targeted therapies has fueled the advancement of new developments for beta and alpha radiation therapy in both the academic medical community and within the pharmaceutical industry. There has been significant recent interest in alpha particles due to their high cytotoxic potential that can be used effectively in cancer therapy. Targeted alpha-particle therapy (TAT) involves the irradiation of cancer cells, micrometastases, or tumors by emitting a single alpha particle or by a cascade of alpha particles within its vicinity.

Alpha particles (H4e)2+ are potent therapeutic effectors that have two advantages over beta therapies. First, the high linear energy (LET) of an alpha particle is two or three orders of magnitude greater than the LET of beta particles (100 keV/µm vs. 0.2 keV/µm). This unique characteristic of alpha particles allows a larger fraction of the total energy to be deposited in the cells, thus increasing therapeutic effectiveness. In sharp contrast, for the application of beta radiation, which has limited linear energy transfer, the cell must be hit by many thousands of beta particles before it is successfully killed [1,2,3,4,5]. In addition, the short-range of alpha radiation in human tissue of 50 to 100 µm (2–3 cell diameters), due to the high LET, compared to that of β particles (maximum ranges of 2 to 11 mm), allows the effective killing of targeted cancer cells due to the highly cytotoxic radiation, while limiting damage to surrounding healthy tissue. Therefore, targeted nanomedicine advances in alpha particles-based delivery offer the best potential for treating micrometastases while sparing normal tissues [6,7,8].

The high linear energy and the short-range features of alpha radiation are reflected in the production of DNA double-strand breaks (DSB), which increase the toxicity due to the limited ability to repair the damage, thus triggering instant cell death from a single atomic decay that passes through the nucleus. The toxicity to the tumor cells from the beta particles is less effective because of the onset of free radicals (indirect DNA damage), allowing a most efficient DNA reparation [9]. This increased biological efficacy of alpha particles is independent of oxygen concentration, dose rate, and the cell cycle stage. Moreover, 1–3 tracks through the nucleus can cause cell death [1,2,3,10,11,12,13,14,15].

Despite the effectiveness offered by the use of alpha particles in cancer therapy, some disadvantages, including limited availability or low production for medical applications, have impeded their widespread use in cancer therapy. Although there are more than 100 radionuclides that decay by alpha emission, most of them have either too short or too long half-lives for therapeutic use, or their chemical properties do not allow their optimal use in medicine. The choice of suitable radionuclide depends on multiple criteria: half-life, decay scheme, recoil energy, associated emission, physical and chemical properties, and availability. Table 1 shows the physical properties of several alpha emitters used in TAT [5]. The most difficult problem with the alpha particle management is associated with the radioisotope daughters release as well as lack of appropriate bifunctional chelating systems affording in vivo stability, a requirement to achieve selective targeting of alpha emitters at tumor sites [5,16]. Currently, targeted therapy using alpha-emitting isotopes presents an opportunity in the development of cancer therapeutic agents for treating various neoplastic diseases.

## 2. Alpha Emitters Used in TAT

Producing alpha emitters implies separating them from natural radionuclides with long half-lives and a combination of alpha emitters that can be generated from either: (a) legacy material, (b) nuclear reactors (neutron irradiation), or (c) accelerators (irradiation with charged particles). After their production, separating the desired radionuclide from the impurities is extremely important [5,16]. Several chemical separation methods, including precipitation, distillation, liquid-liquid extraction, ion exchange, or solid-phase extraction resin separations, can be used to produce radiopharmaceutical-grade alpha emitters for TAT applications [5].

### 2.1. Actinium-225 (^225^Ac)

**Figure 1:**
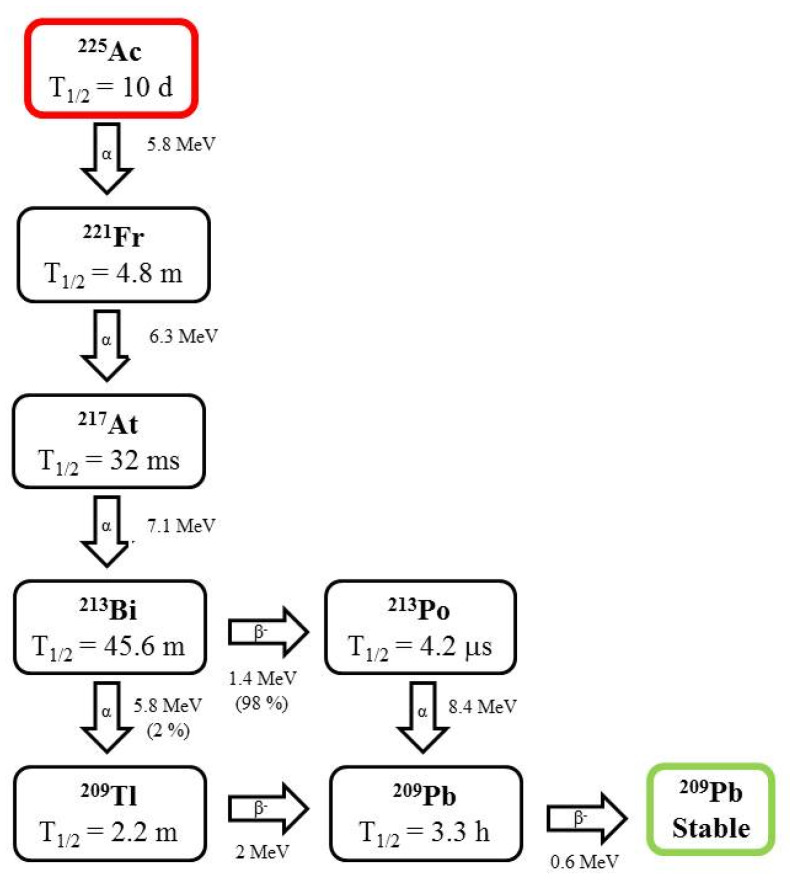
Decay schema of ^225^Ac.

Actinium is considered to be the most potent alpha emitter for use in TAT, since it decays to stable ^209^Bi (t_1/2_ = 1.9 × 10^19^ y) through a series of six daughter radionuclides, with a total emission of four α particles with an energy of 5.8 MeV (total energy of 28 MeV) and associated tissue ranges of 50 to 90 µm (Figure 1) [3,13,17].

^225^Ac can be obtained either from the decay of ^233^U or by proton irradiation of ^226^Ra in a cyclotron through the reaction ^226^Ra(p,2n) ^225^Ac. However, the main source is its parent, ^229^Th (t_1/2_ = 7932 y), which is the first decay product of ^233^U (t_1/2_ = 1.592 × 10^5^ y) and is produced by radiochemical extraction [18]. Currently, there are three ^229^Th sources worldwide that can provide ^225^Ac: Institute for Transuranium Elements (ITU) in Karlsruhe, Germany; Oak Ridge National Laboratory (ORNL), USA; and Institute of Physics and Power Engineering (IPPE) in Obninsk, Russia. The overall production of ^225^Ac from the available ^229^Th sources is approximately 62 GBq per year [2,6].

Actinium isotopes are typically +3 ions with an ionic radius of 112 pm; they can be used as a parent radionuclide for the production of a ^225^Ac/^213^Bi generator or used directly for the radiolabeling of targeting systems [5,19].

The main disadvantages of ^225^Ac are its low availability, recoil energy, and the complicated chemical properties of the daughters that decrease the stability of radiolabeled constructs and represent a risk for in vivo therapeutic applications [19].

### 2.2. Astatine-211 (^211^At)

**Figure 2:**
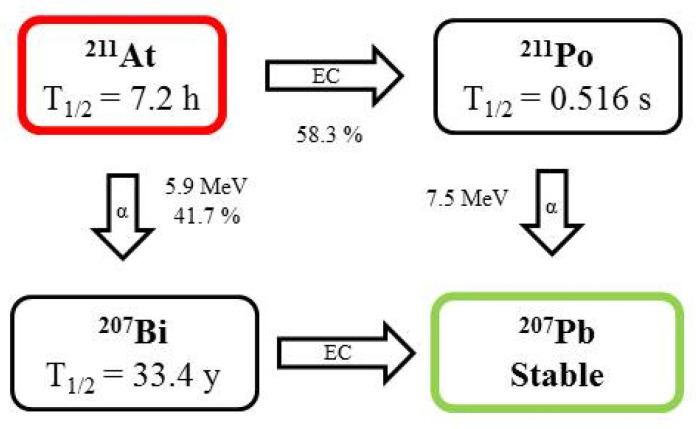
Decay scheme of ^211^At.

Astatine (t_1/2_ = 7.2 h) is a halogen and the only non-metal among the alpha emitters used for therapeutic applications [5]. This radionuclide decays through a branched pathway. The first branch decays to ^211^Po (t_1/2_ = 526 ms), after which it decays to stable ^207^Pb. In the second branch, it decays to ^207^Bi (t_1/2_ = 31.55 y), which then results in stable ^207^Pb after emission of X-rays. The decay of ^211^At is considered 100% alpha due to ^211^Po (the short-lived isotope) (Figure 2) [5,20]. ^211^A is produced in a cyclotron by α particles accelerated to energies of 28–29 MeV bombardment onto a metallic bismuth target via the nuclear reaction ^209^Bi(α,2n)^211^At, followed by simple dry distillation and isolation from irradiated bismuth target. This reaction generates energies between 21 and 40 MeV, with a maximum of about 31 MeV for medical applications [5,16]. An alternative method of ^211^At production is through a ^211^Rn/^211^At generator. The availability of ^211^At at present is limited due to the lack of appropriate cyclotrons. However, the new high-energy cyclotron, ARRONAX (Nantes, France), will contribute to a considerable increase in its production [2].

Astatine can exist in different oxidation states, having similar chemical and radiolabeling properties with heavy halogens such as iodine. The stability of the carbon-halogen bond decreases as we advance within this group in the periodic table, the carbon-astatine bond being the weakest. This bond is known to be sensitive to redox potential, especially in acidic conditions. The astatine release is closely related to the chelator atoms and their ability to stabilize it. In vivo destatination can cause accumulation in the thyroid, lung, stomach, or spleen [21].

Radiolabeling of non-activated aromatic compounds has an inherent disadvantage because such reactions are slow and result in low yields. However, the use of organometallic intermediates facilitates the reaction smoothly with high yields and good radiochemical purity [5,19].

^211^At has great potential to be used in TAT due to its low production cost. However, the main disadvantage is its low availability due to the lack of an accelerator capable of irradiating a target with an alpha-particle beam at 28 MeV [19].

### 2.3. Bismuth-213 (^213^Bi)

**Figure 3:**
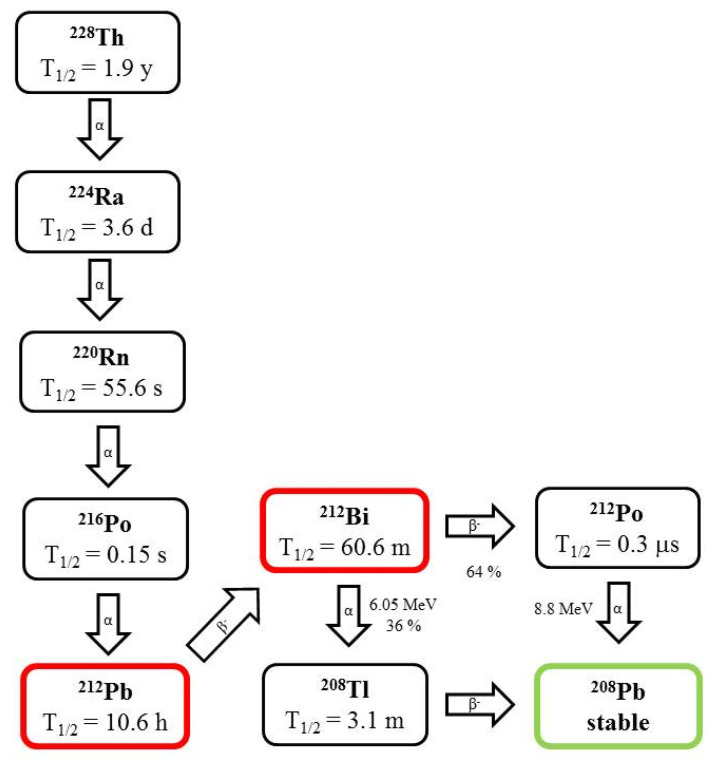
Decay scheme of ^212^Bi and ^212^Pb.

Bismuth (t_1/2_ = 45.6 m) is a mixed alpha/beta emitter that decays to metastable ^209^Bi as a decay product of the ^233^U series. Only 2.14% of ^213^Bi decays via emission of an alpha particle to ^209^Tl with an average energy of 8.35 MeV, while 97.86% disintegrates via beta emission to the pure α emitter ^213^Po (t_1/2_ = 4.2 µs) (Figure 3). ^213^Bi can be eluted from a ^224^Ra generator, owing to their short half-lives. However, the main drawbacks associated with short half-lives are that the entire processing of radiolabeling and quality control must be performed in a very short time [19]. Bismuth is the heaviest stable element in the periodic table with oxidation states of +3 or +5. Its coordination numbers vary from 3 to 9 or 10. Bi^3+^ forms stable complexes with electronegative atoms such as oxygen, nitrogen, sulfur, and halogens [5].

### 2.4. Lead-212 (^212^Pb)

Lead is the father of ^212^Bi and has been used for in vivo generated ^212^Bi. The recoil energy of ^212^Pb is 128 keV (Figure 3) [19].

### 2.5. Radium-223 (^223^Ra)

**Figure 4:**
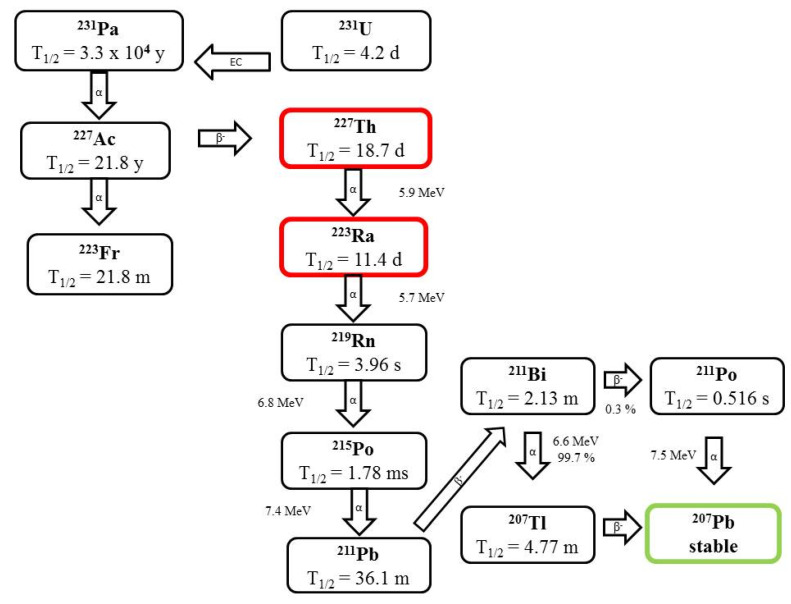
Decay scheme of ^223^Ra.

Radium (t_1/2_ = 11.43 d) decays to stable ^207^Pb under emission of four α particles with energies between 5.7 and 7.4 MeV, releasing total energy of ~28 MeV, and is generated from the decay of ^227^Ac to ^227^Th by the emission of a beta particle followed by alpha emission to produce ^223^Ra (Figure 4) [16]. These radionuclides decay through a cascade of short-lived alpha- and beta-particle emitters to stable lead and bismuth, releasing high total energy of about 28 MeV [22].

Its production for clinical applications involves ^227^Ac and ^227^Th isolation from ^231^Pa. However, the limited availability of the source of ^231^Pa has recently led to the development of alternative production techniques based either on the isolation of the ^227^Ac by-product from the cyclotron production of ^225^Ac or after isolation of ^227^Ac obtained from actinium-227/beryllium-227 generator sources. Radium radionuclides too can be obtained from their long-living parent radionuclides: ^223^Ra from ^227^Ac (t_1/2_ = 21.8 y), ^224^Ra from ^228^Th (t_1/2_ = 1.9 y), and ^225^Ra from ^229^Th (t_1/2_ = 7880 y) [2,19,22]. The main disadvantage of ^223^Ra, apart from their limited availability, is that the recoil energy and different chemical properties of the daughter isotopes decrease the stability of radiolabeled constructs [19].

### 2.6. Thorium-226 (^226^Th)

**Figure 5:**
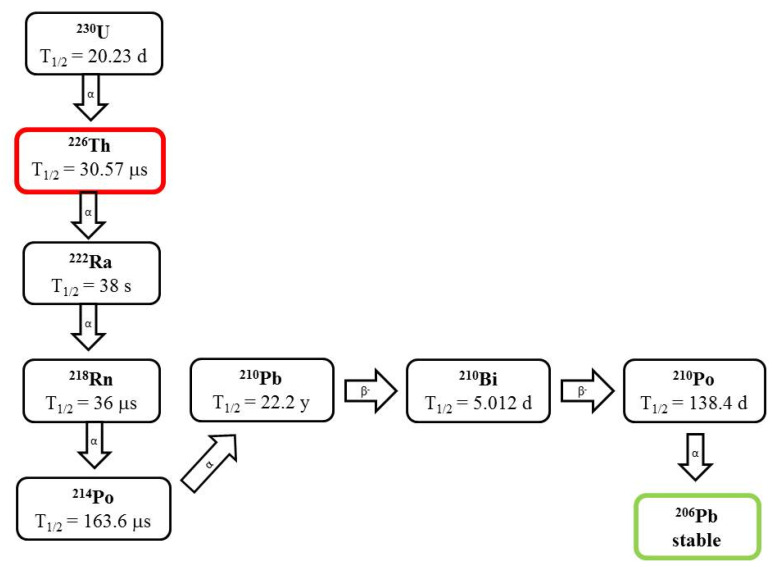
Decay scheme of ^226^Th.

Thorium (t_1/2_ = 30.7 m) is a promising radionuclide for use in targeted α emitter therapy due to the emission of four alpha particles with progressively decreasing half-life. ^226^Th can be eluted from generator systems containing the alpha emitter ^230^U (t_1/2_ = 20.23 d) (Figure 5). Indeed, a successful cyclotron production of ^230^U has been demonstrated via proton irradiation of ^231^Pa according to the reaction ^231^Pa(p, 2n) ^230^U [2,5,23].

### 2.7. Terbium-149 (^149^Tb)

**Figure 6:**
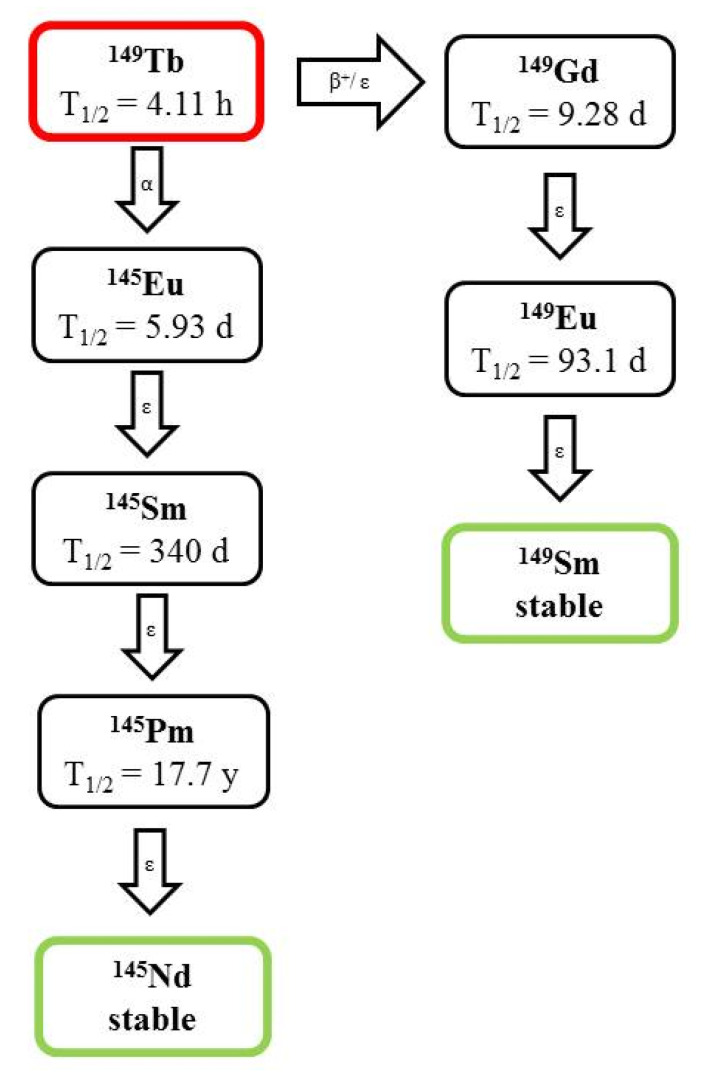
Decay scheme of ^149^Tb.

Terbium (t_1/2_ =  4.118 h) shows a branched decay where only 17% is disintegrated under the emission of an α particle to give rise to ^145^Eu, while 83% decay to ^149^Gd either via β^+^ emission (7%) or electron capture (76%). ^149^Tb has two daughter nuclides, ^145^Sm (t_1/2_ = 340 d) and ^145^Pm (t_1/2_ = 17.7 y) (Figure 6).

The main production route for ^149^Tb is light particle-induced reaction ^152^Gd(p,4n) ^149^Tb (Ep = 50 MeV) [5]. ^149^Tb also has been produced at CERN in Geneva (Switzerland) by irradiation of a tantalum foil target with 1 or 1.4 GeV protons. The radionuclides that are generated during this spallation process are ^149^Tb, ^149^Dy, ^149^Gd, and ^149^Eu and are separated via cation exchange chromatography [2].

## 3. Alpha Emitters in Medical Applications

The high LET of alpha particles gives them an increased relative biological effectiveness (RBE), and therefore, they are capable of destroying tumors causing limited collateral damage to the surrounding healthy tissue. Nevertheless, the use of alpha particle-emitting radionuclides is hampered by the following issues: low availability and high costs, and are too short-lived (one hour or less), making transport of the radionuclides from the place of production to treatment sites highly challenging. Therefore, radionuclides with long half-lives such as ^225^Ac and ^223^Ra are used more frequently in clinics. It is important to recognize that the long-lived α emitters exhibit complex decay chains in which the daughter radionuclides receive high recoil energies (~100 keV) when the α particle is emitted. Therefore, alpha emitters require the use of vectors with high affinity and specificity in addition to maintaining suitable in vivo stability and rapid absorption by target cells. Several candidate isotopes for TAT are currently under preclinical and clinical evaluation, including ^225^Ac, ^149^Tb, ^211^At, ^212^Bi, ^212^Pb, ^213^Bi, ^223^Ra, and ^224^Ra [7,24].

## 4. Recoil Energy

**Figure 7:**
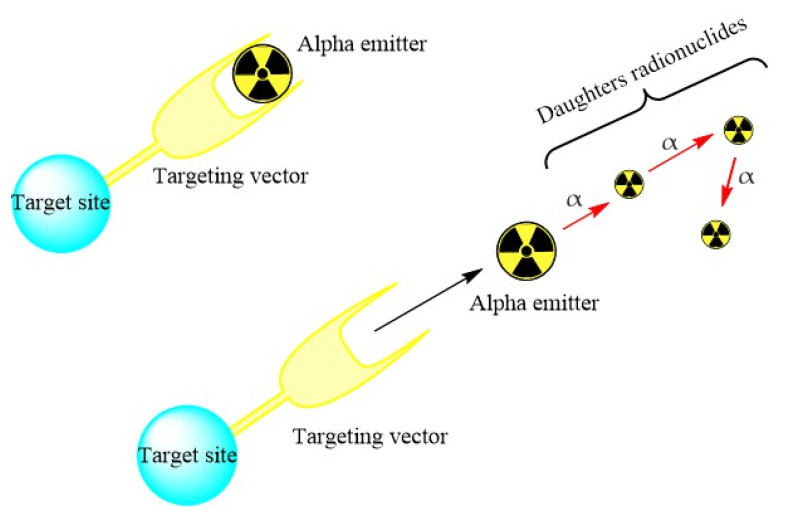
Schematic representation of recoil energy.

Alpha particle use is mainly restricted by recoil energy despite their well-known large cytotoxic effects. The recoil energy causes the breakdown between the targeting molecule or chelate and the radionuclide. This release implies the emission of the first alpha particle and the parent transformation into a new daughter owning different chemistry (Figure 7). Subsequently, there is a recoil of the main atom away from the chelate. Part of the decay energy is transferred to a daughter nucleus due to the momentum conservation law, and subsequent intermediates that emit alpha (daughter) particles have recoil energy due to the daughter nuclide of approximately 100 keV. This has the ability to break at least 10,000 chemical bonds (assuming 10 eV/one bond). This is undesirable because alpha decay will cause bond breakage and the escape of daughter atoms that can target and irradiate healthy organs and tissues, thus reducing the therapeutic dose administered to the diseased site [11,19,23,25,26,27].

The distribution and distance covered by the recoiling daughters through the body depend on the recoil energy, diffusion processes, blood flow, and the location where the recoil atoms are released. Although most of the time, the recoils will be released in the bloodstream, and their fate will be determined by their affinity to organs and tissues provided that they live long enough to reach their biological destination [23,28]. Another problem associated with working on alpha particles is that they are emitted isotropically, and the emission directions of the successive decays in the decay chains are not correlated [23]. For these reasons, parameters such as the biological half-life of the radionuclide and that of its radioactive daughters, the in vivo stability of the carrier, uptake in the reticulum endothelial system (RES), plasma clearance, elimination routes, released activity, the fraction of the radioactive daughter nuclei, and the metabolic fate play an important role and should be taken into account for the in vivo evaluation of the radiopharmaceuticals used for TAT [27].

## 5. Strategies

Alpha particles are extremely effective in killing tumor cells, but they can also cause measurable damage to healthy tissues due to the chemical instability of radiolabeling. Therefore, it is of profound importance to achieve the optimum in vivo stability of the radiopharmaceutical(s) while confining alpha emitters to the tumor microenvironment. To meet these stringent needs, strategies are being developed to promote retention of the decay daughters and reduce recoil energy impact. We discuss here three important strategies: (1) encapsulation of radionuclides in a nanocarrier, (2) use of targeting vehicles (antibodies or peptides), and (3) local administration.

### 5.1. Encapsulation of Radionuclides in a Nanocarrier

This strategy, along with targeted design, facilitates high target accumulation, mainly for radionuclides that present branched decay such as found in ^225^Ac, ^223^Ra, and ^227^Th. This strategy allows encapsulation of radionuclides within a nanocarrier (for example, liposomes, nanoparticles, etc.) that must be large enough to sequester physically all the setbacks of the decay chain in its structure and thus prevent its release and that of the daughter radionuclides escaping from the target site, all aimed at preventing nonspecific radiotoxicity [7,9,11,13,23,24].

### 5.2. Targeting Vehicle

This option consists of developing targeting vehicle molecules such as antibodies or receptor-specific peptides with high-selectivity targeting vectors to facilitate rapid and effective internalization in tumor cells while limiting irradiation of non-target tissues. This strategy has shown clinical potential through both antibodies (pre-targeted radioimmunotherapy (PRIT)) and peptides (peptide receptor radionuclide therapy (PRRT)) [10,20,24,28]. 

### 5.3. Local Administration

Local administration of alpha emitters allows the deposition of radioactivity directly into the target site via injection. It is prudent that the alpha-emitting systems be applied in a low irrigation region to ensure that no daughter radionuclides may infiltrate blood circulation [21,28].

In this review, we provide elaborate details on the first strategy wherein mother radionuclides are encapsulated into a nanoparticle to confine all recoils of their decay chain within its structure. Such a nanoparticle requires a suitable size and structure and should allow an adequate surface functionalization to enable efficient targeting. The success of this approach hinges heavily on the chemical nature and dimensions of nanocarriers. The recoil energies of the daughter nuclides and their displacement range within the material that the nanocarrier is made up of needs to be carefully chosen because the material and size of the nanoconstruct would determine the energy loss of recoils in nanoconstruct materials [16].

There are three important factors to consider when designing a nanocarrier. First, the distribution of daughter recoils is mitigated over time, so their distribution within the organism would also depend on their half-life. Second, daughter recoils distribution is mitigated by size and the chemical constitution of the material of nanocarrier due to the recoiling nuclide consuming some of its energy as it passes through the nanocarrier. Finally, recoils distribution mitigation can be handled by the number of nanocarriers or layers because, even though the recoil ion may escape a nanocarrier or layer, the probability of it entering or slowing down within surrounding nanoconstructs is relatively high [9,27,29,30,31]. In addition, for every subsequent radioactive daughter, the probability of remaining within the physical limits of the liposomal carrier is decreased as compared to the corresponding previous radioactive daughter.

## 6. Chelating Agents

The high energy of α particles and their superior cell killing power demands high stability with the targeting vector, as well as high specificity, good in vivo stability, and fast uptake by target cells. Very long times in circulation, even longer than the life of radionuclides in media, can cause irradiation and nonspecific off-target toxicity [5,9]. It is well known that the main limitation for the application of α emitters in radionuclide therapy is the escape of daughter radionuclides of radiopharmaceuticals since chelating agents (cyclic or linear) are not capable of withstanding the recoil energy of decay daughters (100–200 keV), which is greater than the energy of any chemical bond [16].

In the bonding of a radionuclide(s) with targeting vector(s), the conjugation of such vector(s) with “bifunctional” chelating agents (BCA) is involved. The most common bioconjugation techniques involve peptide coupling reaction between a carboxylic acid and a primary amine, the peptide coupling between activated esters of tetrafluorophenyl (TFP) or *N*-hydroxysuccinimide (NHS), and a primary amine; a thiourea bond formation between an isothiocyanate and a primary amine; a thioether bond formation between a maleimide and thiol; the standard Cu(I) catalyzed Huisgen 1,3-dipolar cycloaddition (“click” reaction) between an azide and an alkyne; and Diels–Alder “click” reaction between a tetrazine and transcyclooctene [32]. BCA, generally, possesses two functional groups, one is a metal-binding moiety (e.g., DTPA or DOTA), and the other is a chemically reactive functional group, capable of binding to a targeting moiety such as peptides or antibodies [19,22,33,34,35]. The structure and physical properties of the radiometal-chelate complex have a great impact on the pharmacokinetic properties of a radiopharmaceutical. Numerous investigations suggest that changing the chelating agent considerably modifies the biodistributions and in vivo stability [32].

Acyclic ligands are more flexible, offering faster chelation kinetics, but they afford lower in vivo stability. Macrocyclic ligands offer rigid structures with facile chelate formations that have higher stability in vivo but with slower kinetics. The ability to quantitatively radiolabel in less time at room temperature is always an attractive property of acyclic chelators, whereas macrocycles often require heating for extended times. Fast room temperature radiolabeling becomes a need when working with bifunctional chelating agents conjugates with heat-sensitive molecules (e.g., antibodies, peptides) or when working with short half-life radionuclides (e.g., ^212/213^Bi) [32].

A description of the most common bifunctional reagents used with alpha-emitting radionuclides is provided in the following sections, and their structures are depicted in Table 2.

### 6.1. ^225^Ac

Larger ligands, HEHA and macropa, have been explored for ^225^Ac for high in vitro and in vivo stability conjugates obtained at room temperature [36,37,38]. Chappell et al. (2000) developed 2-(4-isothiocyanatobenzyl)-1,4,7,10,13,16-hexaazacyclohexadecane-1,4,7,10,13,16-hexaacetic acid (HEHA-NCS) as a chelating agent and radiolabeled three mAbs with ^225^Ac. In this study, ^225^Ac-HEHA showed unstable coordination chemistry and release of the daughter radionuclides in vivo [37]. The HEHA and PEPA macrocycles have been widely used with ^225^Ac, but recently it has been shown that DOTA and derivates have better properties in vivo; however, the high temperatures required for radiolabeling of ^225^Ac with DOTA are very unfavorable for sensitive antibodies [32]. The current method used for synthesizing ^225^Ac-DOTA antibody conjugates employs two steps: first, radiolabeling the p-SCN-Bn-DOTA (BFC) at 60 °C with ^225^Ac; second, the antibody conjugation (thiourea coupling); and finally, the purification steps prior to in vivo utility [37,39,40]. Song et al. (2009) compared the therapeutic effects of ^225^Ac, ^213^Bi, and ^90^Y using an antibody vector and demonstrated ^225^Ac to be the most effective in terms of in vivo characteristics. On the other hand, biodistribution studies of ^221^Fr and ^213^Bi (^225^Ac daughters) revealed accumulation in kidneys and contributed to the long-term renal toxicity [41]. Clinical trials in humans with ^225^Ac-DOTA-HuM195 (humanized anti-CD33 monoclonal antibody) have shown DOTA being the current “gold standard” for ^225^Ac [39,49].

### 6.2. ^212^Pb

DOTA has been used successfully with ^212^Pb; however, slow radiolabeling and low stability were not ideal. Today, the chelator TCMC produced by replacement of the carboxylic acid donor arms of DOTA with amide arms is considered the best chelator available for ^212^Pb [42,44,45].

### 6.3. ^212/213^Bi

The short half-life of ^212^Bi requires rapid radiolabeling kinetics, thus requiring the use of acyclic ligands. Several studies have been reported on the use of derivatives of functionalized diethylenetriamine pentaacetic acid (DTPA) conjugated to mAbs and peptides, including the cyclic DTPA anhydride derivative, 2-(*p*-isothiocyanatobenzyl)-DTPA (SCN-Bz-DTPA), isothiocyanatobenzyl derivative of C-functionalized trans-cyclohexyl DTPA (CHX-A″-DTPA), and 2-(*p*-isothiocyanatobenzyl)-6-methyl-DTPA (Mx-DTPA, 1B4M-DTPA). DTPA complexes have shown good stability in vitro, but only the CHX-A″-DTPA forms suitably stable complexes in vivo. DOTA has also been successfully used for labeling mAbs with ^212^Bi that demonstrated in vivo stability in an animal model. However, the slow kinetics of radiolabeling is considered a disadvantage [5,36].

In a comparative study of various macrocyclic ligands (e.g., NOTA, TETA, and DOTA), DOTA was found to be the most promising ligand since it formed stable complexes with both lead and bismuth [43]. Later, a derivative of DOTA was studied in which the pendant carboxylate arms were replaced by carbamoyl methyl arms, and the macrocyl TCMC or DOTAM emerged. The antibody system with the new macrocyl TCMC had an easier synthesis process than that of DOTA and showed a high radiochemical yield (>95%); in addition, they showed a loss of between 15% and 30% of ^212^Bi from the system [46].

Chong et al. (2008) evaluated the NETA and bifunctional derivatives C-NETA and C-NE3TA. Their results showed good stability with ^212/213^Bi during 11 days in blood serum. Biodistribution studies of C-NETA complexes demonstrated excellent clearance and good stability in vivo [48]. Later, in 2011, Chong et al. showed 3p-C-NETA and trastuzumab immunoconjugates exhibit rapid radiolabeling kinetics and excellent in vitro and in vivo stability [51]. Song et al. (2011) managed to improve the radiolabeling kinetics of ^212/213^Bi with 3p-C-DEPA (derived from DOTA) compared to DOTA, without observing significant changes in in vivo stability. 3p-C-DEPA is today considered to be the “gold standard” for this radionuclide [47].

### 6.4. ^223^Ra

Radiolabeling for ^223^Ra has been developed with DTPA, DOTA, and calix [4] arene tetraacetic acid chelating systems. It has been shown that calix [4] arene provides the most stable complexes, but its stability is not optimal for in vivo use. So far, a vast majority of the ligands used have failed in the development of stable in vivo chelates of ^223^Ra. This is where the application of nanotechnology appears to circumvent this vexing problem. Radiolabeling of ^223^Ra through encapsulating it in different types of nanomaterials is an emerging approach to achieving in vivo stable nanoradiopharmaceuticals of ^223^Ra [5,51,52,53].

### 6.5. ^149^Tb

Non-radioactive terbium has shown high stability when chelated to DOTA, DOTA derivatives, and CHX-A″-DTPA [5]. Beyer et al. (2012) demonstrated in vitro studies of ^149^Tb chelated to conjugated MAb-CHX-A″-DTPA [50]. Recently, in vivo study using four Tb isotopes attached to the same conjugate cm09 (DOTA ligand attached targeting vector) demonstrated their potential for in vivo applications [54]. These results demonstrated that terbium radioisotopes can be readily chelated using CHX-A″-DTPA and DOTA [52].

### 6.6. ^226/227^Th

The antibody labeling with ^227^Th has been carried out using mainly the DOTA chelating agent. The antibody labeling with ^227^Th by a two-step process resulted in 6–7% yields [55]. CHX-A″-DTPA (DTPA derivatives) may be useful for ^226^Th, and DOTA-Bn-NCS has been used for labeling of mAbs with ^227^Th, but the labeling yields were low [52]. Ramdahl et al. (2016) presented an octadentate bifunctional chelator containing hydroxypyridinone (HOPO) moieties. (Me-3,2-HOPO)_4_-Bn-NCS facilitates the labeling of mAbs with ^227^Th in 30 min and at room temperature, producing high radiochemical yields (>96%). Their results showed that (Me-3,2-HOPO)_4_-chelated ^227^Th has good in vivo stability [56].

### 6.7. ^211^At

Labeling of ^211^At with conjugates based on phenyl rings has shown in vivo instability and low labeling yields. Recently, the formation of aromatic boron-astate bonds has been proposed as an attractive alternative to achieve optimum in vivo stability. The advantage of this procedure is that the conjugation to protein and/or antibodies can be performed in the first step, thus reducing the reaction time [5]. Wilbur et al. (2012), in their studies using the closo-decaborate^2−^ moiety (aromatic boron cage moieties), developed immuno-conjugates that can be rapidly labeled (under 2 min) with high radiochemical labeling yields (80–95%) with good in vivo stability [57].

## 7. Targeting Vectors

The successful implementation of α emitters in medicine requires their effective targeting with high selectivity at tumor sites. Targeting vectors, including mAbs, peptides, or nanoparticles (e.g., liposomes, micelles, dendrimers), will play prominent roles in the development of targeted α-emitting radiopharmaceuticals.

There are two main approaches by which a targeting vector can be linked to an α-emitting nuclide. In the first approach, the bifunctional reagent is labeled with an α-emitting nuclide with subsequent conjugation with the tumor-targeting vector. In the other alternative, the bifunctional reagent is first conjugated to the targeting vector, and then the conjugated vector is radiolabeled with an alpha emitter [5]. Using ^211^At, conjugations with vectors such as MAb have been reported [19,58]. In the first clinical trial (phase I/II study), a ^211^At-labeled MAb (^211^At-ch81C6) was evaluated to treat brain cancer. In the second clinical trial (phase I study), ^211^At-labeled MAb (^211^At-MX35F(ab′)_2_) was administered in ovarian cancer patients. Both studies showed that the toxicity was not significant [59,60]. A ^211^At-labeled anti-CD45 conjugate (^211^At-BC8-B10) is being evaluated in phases I/II clinical trials (clinicaltrials.gov NCT03128034 and NCT03670966). ^211^At-BC8-B10 has been administered intravenously to treat patients with acute myeloid or lymphoblastic leukemia and myelodysplastic syndrome [61].

The in vivo generator ^212^Pb/^212^Bi has been evaluated in radiotherapy directed at the human epithermal growth factor receptor (HER2 or HER1). Preclinical studies of trastuzumab conjugated with the bifunctional agent TCMC-Bn-NCS have been carried out for radiolabeling of ^212^Pb. Preclinical studies of trastuzumab conjugated with the bifunctional agent TCMC-Bn-NCS have been carried out for radiolabeling of ^212^Pb (^212^Pb-TCMC-trastuzumab) evaluating the system alone or in combination with drugs such as paclitaxel, gemcitabine, etc. for treatment of ovarian, prostate, and pancreatic cancer [62,63]. TCMC-Bn-NCS have been conjugated with different antibodies. In 2016, Milenic et al. evaluated the biodistribution, pharmacokinetics, and toxicity of ^212^Pb-TCMC-trastuzumab in a phase I clinical trial in patients with HER2-expressing ovarian cancer. The results showed that the redistribution of radionuclide was minimal and the myelosuppression was not significant [63], Later, in 2018, Milenic et al. evaluated the conjugation of panitumumab F(ab′)_2_ with TCMC as a targeting agent for epidermal growth factor receptor [64]. 

In addition, preclinical and clinical trials with different monoclonal antibodies (mAbs) and peptides labeled with ^213^Bi for treating leukemia (^213^Bi-HuM195), lymphoma (^213^Bi-anti-CD20 MAb), melanoma (^213^Bi-9.2.27), bladder cancer (^213^Bi-anti-EGFR MAb), glioblastoma (^213^Bi-substance P), and neuroendocrine tumors (^213^Bi-DOTATOC) have been reported [64,65,66,67,68,69], and ^225^Ac-labeled mAbs and peptides for acute myeloid leukemia (^225^Ac-DOTA-NCS with lintuzumab or ^225^Ac-HuM195mAb), for prostate cancer (^225^Ac-PSMA-617), glioma (^225^Ac-Substance P), and neuroendocrine tumors (^225^Ac-DOTATOC) have been evaluated [13,18]. In clinical trials, hundreds of patients with prostate cancer have undergone the treatment with ^225^Ac and ^213^Bi-labeled PSMA-617 and have presented excellent responses [67,70].

Hagemann et al. in 2016 evaluated ^227^Th-labeled anti-CD33 antibodies for the treatment of acute myeloid leukemia in vitro and in vivo [71] and later in 2017 evaluated ^227^Th-labeled anti-CD70 to immunotherapy [72]. Two clinical trials (clinicaltrials.gov NCT02581878 and clinicaltrials.gov NCT03507452) have been reported. The first was to evaluate an anti-CD22 MAb conjugate, ^227^Th-epratuzumab (BAY1862864), to establish the dose in patients with CD22 positive non-Hodgkin’s lymphoma (NHL), and in the second trial, a ^227^Th-labeled MAb conjugate (BAY2287411) was used to evaluate the safety and maximum dose in patients with epithelioid malignant mesothelioma and ovarian cancer [5]. 

## 8. Nanoradiopharmaceuticals Based on Alpha Emitters

In the continued quest and exploration of nanoparticles as drug carriers, several approaches have been developed to load nanoparticles with α-emitting radionuclides to dissipate the recoil energies of the daughter radionuclides in the nanoparticles and to avoid their release. Thus far, zeolites, liposomes, polymersomes, and metal-based particles have been studied to retain the recoil energy of alpha-emitting radionuclides and are presented in Table 3.

Nanoparticle-based systems have been designed to improve biodistribution, stability, specificity, pharmacological and targeting properties, and daughter retention, as well as to exploit the theranostic potential for dual imaging and therapy.

Inorganic nanoparticles (NPs) made of lanthanide phosphate, zeolites, magnetite, hydroxyapatite, barium sulfate, and calcium carbonate have shown high encapsulation of alpha emitters, while decay daughters have been partially retained. Kucka et al., in 2006, prepared ^211^At-radiolabeled silver nanoparticles and coated the surface with poly(ethylene oxide) polymer. The radiolabeling yields were higher than 95%, but the extent of retention in tumors was not reported [73]. In 2011, Woodward et al. developed inorganic lanthanum phosphate nanoparticles to incorporate ^225^Ac [^225^Ac(La(^225^Ac)PO_4_)] conjugated with the monoclonal antibody mAb201B. Results showed almost complete retention of ^225^Ac into nanoparticles; however, 50% of the daughter nuclides (^221^Fr and ^213^Bi) were released in in vitro investigations. In vivo release of ^213^Bi in lungs, liver, spleen, and kidney, indicating some release of this daughter nuclide following nuclear recoil, was observed despite its encapsulation in the nanoparticle [74]. Rojas et al. have investigated core + 1 shell and core + 2 shells of LaPO_4_ nanoparticles (NPs with two layers of cold LaPO_4_ deposited on the core surfaces) containing either ^223^Ra or ^225^Ra/^225^Ac to evaluate their ability to retain the parent isotopes and associated daughter products in vitro. They showed that the addition of cold LaPO_4_ shells to the NPs surfaces significantly improves the retention of the daughters’ nuclides. The core + 1 shell retained 88% of ^223^Ra over 35 days. However, in the core + 2 shells NPs, the retention of ^223^Ra and its daughter (^211^Pb) was improved to 99.9% over 27 days. Additionally, the retention of ^225^Ra/^225^Ac parents was approximately 99.9% and 80% for the ^221^Fr and ^213^Bi daughters, respectively, over 35 days [75]. In 2013, Piotrowska et al. synthesized NaA nanozeolite (30–70 nm), in which the percentage of absorption for ^224^Ra and ^225^Ra was above 99.9%. ^224^Ra-nanozeolite showed high stability in physiological and PBS solutions for up to 24 h, while ^225^Ra-nanozeolite was stable up to 2 days. After this time, the leakage of ^224,225^Ra from the nanozeolites was below 0.5%. In contrast, labeled nanozeolites were unstable in EDTA and cysteine solutions [22]. Nanozeolite carriers were also functionalized with silane-PEG-SP and labeled with ^223^Ra. The synthesis rendered 50–80 nm nanoparticles. The leakage of ^223^Ra was less than 0.5%, and the release of ^211^Pb and ^211^Bi (decay products) was 2–5% in human serum, which corresponds to 90% to 95% retention of the decay products [76]. McLaughlin et al. demonstrated that multilayered nanoparticles (NPs) {La_0.5_Gd_0.5_}PO_4_@GdPO_4_@Au doped with ^225^Ac (diameter 27 nm) managed to retain 99.99% of the ^225^Ac within 3 weeks and 88% of the decay products (^221^Fr and ^213^Bi) in studies in vitro. The in vivo behavior showed that ^225^Ac was predominantly present in the liver and spleen, while retention of ^213^Bi (the breakdown of ^225^Ac in NPs) in the liver and spleen after 24 h was 84–63% [31]. Table 3.

**Table 3:** Summary of encapsulation of alpha emitters experiments reported in the literature.

Radionuclide	Nanoparticle (Type)	Retention/Release of Daughter Nuclides	Labeling Yield	Size	Ref
^225^Ac	^225^Ac(La(^225^Ac)PO_4_)	Retention ~100% of ^225^Ac Retention ~80% of ^213^Bi (daughter of ^225^Ac decay) Retention ~50% of the daughters (^221^Fr and ^213^Bi; 1 month) Release 50% of the daughter nuclides ^221^Fr and ^213^Bi in vitro	66%	3–5 nm	[74]
	Multilayered {La_0.5_Gd_0.5_}PO_4_@GdPO_4_@Au doped with ^225^Ac	Retention ~100% of ^225^Ac (3 weeks) Retention of 88% ^221^Fr and ^213^Bi (decay products) Retention of 84–63% of ^213^Bi (breakdown of ^225^Ac in NPs) in liver and spleen after 24 h in vivo	~76%	27 nm	[31]
	Multilayer {La_0.5_Gd_0.5_}(^225^Ac)PO_4_@4GdPO_4_ shell@Au	Retention >99.9% ^225^Ac (3 weeks) Retention in vivo of ^213^Bi (daughter of ^225^Ac decay) in lung tissue > 70% (1 h) and approximately 90% (24 h) ^221^Fr retention ~60% (3 weeks)	---	---	[77]
	Multilayer {Gd_0.75_La_0.25_}(^225^Ac)PO_4_@4 LaPO_4_ shell@Au	Retention ~100% of ^225^Ac (3 weeks) Retention of ^221^Fr 60–89%	76%	19.9 ± 6.5 nm	[78]
	Gadolinium vanadate (GdVO_4_)	Leakage of ^221^Fr (1^st^ daughter of ^225^Ac) and ^213^Bi (3rd daughter of ^225^Ac) was 55.4 ± 3.6%, and 25.6 ± 1.5%, respectively (5 days) Leakage of ^221^Fr and ^213^Bi was of 41.1 ± 2.5% and 19.8 ± 1.16%, respectively (11 days) Leakage of ^223^Ra 73.0 ± 4.0% (4 days), and 30.0 ± 1.7% (33 days) The leakage of ^211^Pb was 44.8 ± 3.1% (35 days)	---	---	[24]
	Gadolinium vanadate core (Gd(^225^Ac)VO_4_) and core + 2 shell (Gd(^225^Ac)VO_4_/2GdVO_4_)	Leakage of ^225^Ac was 15% and 2.4% from core and core + 2 shells Leakage of ^227^Th was 3% and 1.5%, from core and core + 2 shells, respectively Leakage of ^221^Fr (first decay daughter) from core was 40.6 ± 2.4% (2 days) and ~69.5 ± 4.9% (23 days) Leakage for ^213^Bi from core and core + 2 shell was 22.5 ± 1.3% and 19.6 ± 1.9%, respectively Leakage of ^227^Th was ~3% (15 days) and 1.6 ± 0.3% (18 days) Leakage of ^223^Ra from core was 10.0 ± 0.7% (1 day) and 39.0 ± 2.2% (2 weeks)	Yield of ^225^Ac was 77.1 ± 13.2% and 96.6 ± 1.6% for core and core + 2 shell, respectively Yield of ^227^Th was 62.9 ± 2.7% and 81.9 ± 1.2% for core and core + 2 shell, respectively	3.6 ± 0.9 nm for core and 4.4 ± 1.0 nm for core + 2 shell	[79]
	Gd_0.8_Eu_0.2_VO_4_ core and core + 2 shells	The leakage of ^221^Fr from core was 40.9 ± 0.3% (2 days), 55 ± 3.6% (10 days) and 67.6 ± 3.3% (28 days) Leakage of ^221^Fr from core + 2 shells was 36.3 ± 6.2% (10 days) and 45.5 ± 3.6% (28 days) The leakage of ^213^Bi was <15% for core and ~22% for core + 2 shells	Yield core was 41.1 ± 16.5%, shells increased the yield up to 55%	6.1 ± 1.4 nm for core and 12.4 ± 2.0 nm for core + 2 shells	[80]
	Liposomes: Pegylated liposomes membrane charge (zwitterionic and cationic)	^213^Bi retention was 10% (650 nm) ^225^Ac retention in the zwitterionic liposome was ~88% (30 days) ^225^Ac retention in cationic liposomes was 54%	6.4–10.0%	200/400/650 nm	[11]
	Liposomes	^225^Ac retention of 81 ± 7%	55–73%	121 ± 6 nm	[81]
	Liposomes	Retention of ^225^Ac with up to three ^225^Ac nuclides per every 2 liposomes	58.0–85.6%	107 ± 2 nm	[82]
	Multivesicular liposomes (MUVELs)	Retained 98% of ^225^Ac (30 days) Retention of ^213^Bi was 31% (758 nm)	---	---	[83]
	Polymersomes	Retention 221Fr was 71.5% (800 nm, 24 h) Retention 213Bi was 74% (800 nm, 24 h)	^213^Bi was 83 ± 75% (100 nm) ^225^Ac was 67 ± 0.8% (100 nm)	100, 200, 400, and 800 nm	[28]
	Double-layered polymersomes	Retention of ~100% of ^221^Fr (800 nm) Retention of ^213^Bi (recoil atoms) was 80% (800 nm)	---	300–800 nm	[26]
	Polymersomes	Retention of ^225^Ac was 92 ± 3% Retention of ^221^Fr (first daughter) was ~20% Retention ^213^Bi was ~10%	89 ± 0.6%	100/200/400/800 nm	[84]
	Polymerosomes	---	>90% for ^111^In and >64% for ^225^Ac	---	[85]
	Polymerosomes	^221^Fr and ^213^Bi were retained at the tumor site 88 ± 9% and 89 ± 2%, respectively (2 days p.i.)	54–59%	97 ± 37 nm	[86]
	Fullerenes: ^225^Ac metallofullerene	---	---	---	[87]
	TiO_2_	Retained >95% of ^225^Ac and ^221^Fr (10 days)	99.8 ± 2.1%	25 nm	[88]
	Carbon nanotubes	Clear rapidly and shown to be therapeutically effective in vivo	---	---	[89]
	Carbon nanotubes	---	~95%	---	[90]
	Lipid vehicle	^225^Ac were retained to significant extents by during 6 h	62.7 ± 14.6%	106 ± 4 nm	[91]
^223^Ra	LnPO_4_ core and core + 2 shells NPs (Ln = La, Gd)	The core LaPO4 NPs retained up to 88% of ^223^Ra (35 days) Retention of 99.9% of ^223^Ra and ^211^Pb (daughter of ^223^Ra) the core + 2 shells (27 days) Retention ~100% of ^211^Pb (decay daughter) Retention of 99.98% of ^225^Ra/^225^Ac parents and ~80% for the ^221^Fr and ^213^Bi daughters in the core + 2 shells (35 days)	91% for LaPO4 core + 2 shell	3.4 nm for core and 6.3 nm for core + 2 shells	[75]
	Hydroxyapatite (HA)	No significant release of activity was detected	>95%	15 nm	[92]
	Hydroxyapatite (*n*HAp) and titanium dioxide (*n*TiO_2_)	The sorption efficiency on *n*HAp was 95 ± 5% and approximately 100% in the case of *n*TiO_2_	~95%	---	[93]
	Hydroxyapatite (HAP)	---	98%	Width up to 100 nm and length up to 500 nm	[94]
	Hydroxyapatite (HAP)	---	98%	900–1000 μm	[95]
	Liposomes	^223^Ra retention was 96 ± 1% (24 h) and 93 ± 2% (100 h) ^228^Ac retention was 95 ± 2% (24 h)	^223^Ra was 78 ± 6% ^228^Ac was 61 ± 8%	---	[96]
	Liposomes: Pegylated liposomal doxorubicin (PLD)	Liposomal ^223^Ra was relatively stable in vivo and was largely retained in liposomes	51–67%	80 nm	[97]
	^223/224/225^Ra-nanozeolite (NaA)	The leakage in PBS solutions of ^224,225^Ra-nanozeolites was below 0.5%	99.9%	30–70 nm	[22]
	^223^Ra-labeled nanozeolite	The leakage of ^223^Ra was <0.5% Release of ^211^Pb and ^211^Bi (decay products) was 2% (1 day) and 5% (6 days) in human serum Retention of 90–95% of ^211^Pb and ^211^Bi (decay products)	99.9%	50–80 nm	[76]
	BaSO_4_	---	20%	140 nm	[98]
	Superparamagnetic iron oxide nanoparticles Fe_3_O_4_ SPIONS	PBS, bovine plasma, and serum showed a cumulative release of ^223^Ra below 5%	85–99% (PBS)	4–26 nm	[99]
	Reduced graphite oxide	Desorption in PBS + BSA solution <5% of ^99m^Tc Desorption in BSA in PBS <10% of ^99m^Tc Desorption in PBS + BSA solution in 1 h raised to 35% for ^90^Y	Sorption was 10% for ^223^Ra, 90% of ^99m^Tc, 80–100% for ^207^Bi and ^90^Y	4–6 nm	[100]
	Polyoxopalladates (Pd-POM): [^224^Ra]Na-a(Ra)Pd_15_	^224^Ra was incorporated into [^224^Ra] Na-Ba(Ra)Pd_15,_ but the insertion is not selective (lead and bismuth Pd-POM can also be generated)	---	---	[101]
	Calcium carbonate microparticles	Retention >95% for ^224^Ra and its daughter nuclide ^212^Pb (1 week)	>80% of ^224^Ra and ^212^Pb (daughter nuclide)	---	[102]
^211^At	Silver core coated by PEO shell	---	50–97%	18.3–34.7 nm	[73]
	Ultrashort nanotubes	Retention was 19.6% and 11.0% for water and methanol, respectively	77.7–91.3%	20–50 nm in length and 1 nm diameter	[103]
	Gold nanoparticles (AuNPs)	---	>99%	5 and 15 nm	[104]
	Gold nanoparticles (AuNPs)	---	>99%	5 nm	[105]
^212^Pb	Liposomes	---	75%	---	[106]
	Liposomes	^212^Pb and ^212^Bi were 95% retained (20 h)	90 ± 2%	---	[107]
	Hydroxyapatite (HAP)	---	---	---	[108]

The {Gd_0.75_ La_0.25_}(^225^Ac)PO_4_@4 LaPO_4_ shell@Au nanosystem showed 100% of ^225^Ac retention over three weeks. The ^225^Ac retention did not do not show variation compared with {La_0.5_Gd_0.5_}PO_4_@GdPO_4_@Au doped with ^225^Ac, but ^221^Fr retention varied from 60–89% as a function of time, the number of layers, and nanoparticle composition [78]. Later, in 2014, presented the second-generation layered nanoparticles {La_0.5_Gd_0.5_}(^225^Ac)PO_4_@4 GdPO_4_ shell@Au, the ^225^Ac retention was almost 100%; however, the ^221^Fr retention was only 60% in in vitro studies. This second generation achieved more than 70% of ^213^Bi retention in the lungs and approximately 90% at 1 and 24 h after injection [77]. The authors concluded the ability to retain the ^225^Ac and its decay daughters in the NP core depends on its composition and the type and number of added shells [31,79,80]. 

Edyta et al. (2018) proposed titanium dioxide (TiO_2_) nanoparticles (25 nm diameter) as ^225^Ac carrier and their decay products. NPs retained more than 95% of ^225^Ac and ^221^Fr over 10 days in PBS and physiological salt and with a yield of 99.8 ± 2.1% [90]. In 2020, Suchánková et al. studied ^223^Ra sorption on hydroxyapatite (*n*HAp) and titanium dioxide nanoparticles (*n*TiO_2_). The sorption efficiency on *n*HAp was 95 ± 5% and approximately 100% in the case of *n*TiO_2_ [95].

In 2014, Kozempel et al. evaluated the in vitro stability and labeling yield hydroxyapatite nanoparticles (HA-NPs) radiolabeled with ^223^Ra. In their studies, no significant release of activity was detected on stability tests, probably due to resorption of released ^223^Ra and ^211^Pb or due to the decay of the daughter nuclides of short-lived. The labeling yield of ^223^Ra was greater than 95% for intrinsically labeled but did not provide data on the release or retention of daughter nuclides [94]. Later, in 2016, Vasiliev et al. synthesized hydroxyapatite nanoparticles with a width of up to 100 nm and a length of 500 nm, as carriers of ^223^Ra with a yield of up to 98% [96]. Kukleva et al., in 2006, studied the uptake of ^223^Ra in the superparamagnetic nanoparticles (SPION) of Fe_3_O_4_ (diameters between 4 and 26 nm). The in vitro results showed yields greater than 85% in PBS, and the stability tests in PBS, bovine plasma, and serum showed a cumulative release of ^223^Ra below 5% [101]. Westrøm et al., in 2018, proposed calcium carbonate microparticles as carriers of ^224^Ra. The results show that both ^224^Ra and its daughter nuclide ^212^Pb were adsorbed with yields greater than 80% and reported retention of more than 95% for both radionuclides for up to 1 week in vitro. Biodistribution studies showed that the highest activity of ^212^Pb (^224^Ra daughter with the longest half-life) was found in the kidneys, mainly [104]. Reissig et al. (2019) proposed functionalized BaSO_4_ nanoparticles as carriers to ^224^Ra. The NPs had sizes of approximately 140 nm, and it was possible to incorporate 20% of ^224^Ra into the NPs [100]. Gott et al. proposed the use of polyoxopalladates (Pd-POM), in which Ra^2+^ can be easily introduced into the nucleus. The results showed that ^224^Ra was incorporated into [^224^Ra] Na-Ba(Ra)Pd_15,_ but the insertion is not selective, as lead and bismuth Pd-POM can also be generated [103]. Toro-González et al., in 2020, synthesized gadolinium vanadate (GdVO_4_) NPs sizing <100 nm as platforms for ^225^Ac, ^223^Ra, and ^227^Th encapsulation. They reported the leakage of ^221^Fr (1st daughter of ^225^Ac) and ^213^Bi (3rd daughter of ^225^Ac) as 55.4 ± 3.6% and 25.6 ± 1.5% after 5 days in dialysis, and after 11 days it decreased to 41.1 ± 2.5% and 19.8 ± 1.16%, respectively. In the case of ^223^Ra and ^211^Pb (decay daughters of ^227^Th), they showed that the leakage of ^223^Ra (1^st^ decay daughter) reached a maximum of 73.0 ± 4.0% after 4 days, and then decreased to 30.0 ± 1.7% at 33 days finally in ^211^Pb (3rd decay daughter of ^223^Ra). The leakage of ^211^Pb showed that the leak increased from 11.1 ± 1.0% to 44.8 ± 3.1% after 35 days on dialysis [24].

There have been extensive theoretical and experimental investigations on the possibility of using liposomes for testing the retention of recoils in the decay chains of ^225^Ac [11,83,84,85,98] and of ^223^Ra [98,99]. Sofou et al. (2004) prepared pegylated liposomes of different membrane charges (zwitterionic and cationic) and sizes to entrap ^225^Ac with mean encapsulation efficiency between 6.4% and 10.0%. This efficiency translated into 10–40 actinium atoms per liposome. In this research, the theoretical and in vitro fraction of ^213^Bi retention as a function of liposome size and composition was evaluated. Theoretical calculations have revealed that for satisfactory ^213^Bi retention (>50%), liposomes of relatively large sizes (>650 nm in diameter) are required. Experimentally, several studies have verified ^213^Bi retention to be liposome size dependent. The ^213^Bi retention was significantly higher in the 800 versus the 100 nm zwitterionic liposomes. However, not even the mother radionuclide ^225^Ac was completely retained, and ^213^Bi retention was 10% for the largest liposomes (650 nm). Regarding the composition of the liposomes, ^225^Ac retention in the zwitterionic liposome fractions was more than 88% over 30 days. While in cationic liposomes, ^225^Ac retention was above 54%. The authors proposed that the liposomal encapsulation of ^225^Ac to retain the daughters is size dependent [11]. Multivesicular liposomes (MUVELs) were prepared by Sofou et al. in 2007 to increase daughter retention using the MUVELs, which are large liposomes with entrapped smaller lipid vesicles (rigid membranes composed of diheneicosanoyl phosphocholine and cholesterol) containing ^225^Ac. PEGylated MUVELs retained 98% of encapsulated ^225^Ac for 30 days. Retention of ^213^Bi (the last daughter) was 31% in the MUVELs, with an average size of 758 ± 287 nm. As in the previous work, the authors did not observe higher daughter retention attributable to the larger size of the carrier (1 µm diameter). Due to the limitations of stable intact liposomes having such large diameters, it is important to evaluate new materials to develop in vivo stable outer large shell encapsulations [85]. Henriksen et al. (2004) prepared liposomes containing ^223^Radium and ^225^Ac, and the stability and the retention of these radionuclides in serum were evaluated. The loading of ^223^Radium within liposomes was 78 ± 6% with retention of 96 ± 1% after 24 h and 93 ± 2% after 100 h. The loading of ^228^Ac (^225^Ac was not available at the time of the experiments) was 61 ± 8%, and the retention was 95 ± 2% after 24 h [98]. Later, in 2008, Chang et al. (2008) proposed various conditions for loading high activities of ^225^Ac using pegylated liposomes of 1,2-dinonadecanoyl-sn-glycero-3-phosphocholine (DNPC) and cholesterol after showing in a previous study that liposomes can stably retain encapsulated ^225^Ac for long periods. Liposomes were encapsulated with three ^225^Ac nuclides per every two liposomes with a diameter of 121 ± 6 nm, a loading efficiency of 55–73%, and ^225^Ac retention of 81 ± 7% of the initially encapsulated radioactivity was achieved. The main release of ^225^Ac from all liposomes can retain and remain encapsulated for 30 days. The study showed that ^225^Ac is loaded to a higher yield in preformed liposomes and is more stably retained over time when the ionophore liposomes were loading with citrate buffer [83]. Bandekar et al. reported anti-PSMA liposomes with 107 ± 2 nm of diameter loaded with ^225^Ac to selectively kill prostate-specific membrane antigen (PSMA) expressing cells. The loading efficiency of ^225^Ac into liposomes ranged from 58.0% to 85.6%. The targeted and un-targeted PSMA liposomes exhibited similar retention of encapsulated ^225^Ac with up to three ^225^Ac nuclides per every two liposomes. However, their study did not report the retention of ^225^Ac or any of its daughter radionuclides [84]. Jonasdottir et al. in 2006 reported the first phase II study of alpha-emitting radioliposomes with a loading efficiency of between 51% and 67% of ^223^Ra in pegylated liposomal doxorubicin (PLD), which had an average diameter of approximately 80 nm. The comparison between liposomal ^223^Ra versus free cationic ^223^Ra showed that the liposomal ^223^Ra was catabolized, and the ^223^Ra released was either excreted or absorbed by the skeleton. This skeletal uptake increased up to 14 days after treatment, but ^223^Ra in bone was lower in the group given liposomal ^223^Ra versus cationic ^223^Ra, and spleen and bone received the highest absorbed doses. The authors concluded that liposomal ^223^Ra was relatively stable in vivo and that ^223^Ra was largely retained in liposomes [99]. Henriksen et al. (2003) incorporated ^212^Pb into preformed liposomes and evaluated the in vitro retention of it and its radioactive daughter ^212^Bi during incubation in serum, the yield of ^212^Pb within the liposomes was 90 ± 2% after 30 min. ^212^Pb and ^212^Bi were 95% retained in the liposomes after 20 h of incubation [108]. 

Kruijff et al. succeeded in synthesizing [^225^Ac]InPO_4_ nanoparticles by coprecipitation within the polymersomes, with a loading efficiency of 89 ± 0.6% and retention of ^225^Ac of around 92 ± 3%, while retention of ^221^Fr (first daughter) was approximately 20%, and ^213^Bi retention of approximately 10% was reported [86]. Wang et al. evaluated the percentage of recoil daughters that are retained in polymersomes of different sizes upon encapsulation of ^225^Ac and ^213^Bi. Results showed a loading efficiency of 83 ± 5% and 67 ± 0.8% for ^213^Bi and ^225^Ac, respectively, in polymersomes of 100 nm. The highest experimental retention values for ^221^Fr (71.5%) and ^213^Bi (74%) were achieved in the largest polymersomes (800 nm), 24 h after loading. These results indicate that larger polymersomes are needed to attain satisfactory retention of recoiling radionuclides [28]. Thijssen et al. (2012) carried out a study based on computer simulations (Monte Carlo simulations code “NANVES”) describing the best polymersome design for the retention of the recoil daughters of ^225^Ac (^221^Fr and ^213^Bi). Simulations showed that the best polymersome design comprises a double-layered vesicle with the nuclide encapsulated inside the innermost layer and that the size is also important due to larger polymersomes retaining recoils better. For instance, double-layered vesicles with a diameter of 800 nm are capable of retaining ^221^Fr completely and approximately 80% of the ^213^Bi recoil atoms, while the escape of ^213^Bi is reduced to 20% and the percentage of alpha particles emitted from escaped daughter products outside the nanocarrier is less than 10%. The double-layered polymersomes with outer diameters of 300 and 400 nm retained 41% and 47% of the ^213^Bi recoils, respectively [26]. Kruijff et al. (2018) demonstrated polymersomes radiolabeling efficiencies > 90% for ^111^In and > 64% for ^225^Ac. The distribution of the daughter atoms in vivo was not assessed [87].

There also investigations where carbon nanomaterials have been proposed for ^225^Ac, ^223^Ra, and ^211^At radionuclides [89,91,92,102,105]. Gold nanoparticles conjugated to ^211^At [105,106], liposomes with ^212^Pb/^212^Bi [107,108], and hydroxyapatite nanoparticles [109] for retention of ^212^Pb have been proposed with limited success.

## 9. Discussion

Targeted alpha therapy to this day remains a very promising therapeutic option for cancer due to the localized cell destruction that originates from high LET and short ranges of alpha particles. However, achieving complete retention of the decay daughters of some alpha emitters remains the biggest challenge to be solved. The main strategies in targeted therapies to limit the nuclear recoil effects are focused on the use of nanocarriers that can stop the propagation of recoil and provide a controlled release. In this way, the effect of radiation exposure to healthy tissues by free daughter radionuclides formed can be significantly reduced. The nanosystems presented in this review provide an account of the potential of the retention and behavior of radionuclides within nanocarriers, and their performance depends on the different materials used. In general, small nanoparticles usually have better biodistribution and faster clearance than bigger ones [23], but in some cases, in systems such as liposomes and polymersomes, where the retention of decay daughters was measured, they demonstrated size retention effects [11,28,83,84,85,86,87,98,99]. Although the larger sizes perform better in terms of retention of decay daughter, the larger sizes pose severe problems as sizes beyond 150 nm are ineffective in penetrating tumor vasculature and are also less efficient extravasation. Therefore, a trade-off in liposomal size and retention power is important in enhancing retention effects and enhanced patency in the uptake of tumors. In the case of inorganic nanoparticles, it was shown that small nanoparticles can safely confine the decay daughters that occur in a cascade of alpha particles. Strategies suggested by Woodward et al. (2011) [76] and McLaughlin et al. [31,79,80], using small core nanoparticles that are loaded with alpha emitters and then surrounded by confining shells preferentially with high-Z materials, appear to be the most promising.

However, it is still challenging to make accurate predictions on the size and thickness of surrounding shells.

Although some systems show better retentions in vitro than others, the characteristics that the nanocarrier system must meet to completely contain the children of the disintegration chains have not been found to satisfactory outcomes or predictability. Therefore, more detailed investigations are imminent to gain insights on the most optimum nanomaterials capable of providing optimum retention of alpha emitters for ultimate utility in oncology.

## 10. Conclusions

In this review, we have summarized the latest advances in targeted alpha-particle therapy (TAT) through relevant and pertinent literature citations. We have discussed promising results in terms of various systems showing optimum in vitro retention, with limited success so far in achieving optimum in vivo characteristics of TAT. We have provided a strong and scientifically justifiable rationale for the application of nanotechnology in transforming targeted alpha-particle therapy optimally effective in cancer therapy. In this context, we have discussed the importance and the interrelationships of the chemical nature, size, and types of nanomaterials (singular carbon-based, metallic vs. liposomal) on the overall in vivo efficacy of targeted alpha-particle therapy. The data to date suggest that the retention and confinement of alpha-emitting radionuclides do not always depend on the size of nanoparticulate systems. It is important that the nanoparticulate-embedded alpha emitters exhibit sizes within the 50–150 nm range to be able to penetrate the tumor vasculature more effectively. Inorganic core nanoparticles loaded with alpha emitters and surrounded by confinement layers seem to be the most efficient alternative, although more research is needed to find the appropriate thickness of the confinement layers, thus opening up future new research endeavors in TAT.

## Figures and Tables

**Table 1:** Physical properties of main alpha emitters for TAT.

Nuclide	t_1/2_	Main Emissions	Energy (MeV)	Energy Recoil (KeV)	Range (µm)	Daughters	t_1/2_	Decay	Energy (MeV)
^227^Th	18.7 d	α	6	---	50–70	^223^Ra			
^225^Ac	10 d	α	5.8	---	50–90	^221^Fr	4.8 m	A	7
								γ	0.218
				105		^217^At	32.3 ms	A	7
				116		^213^Bi	45.6 m	A	6
								β^−^	0.444
								γ	0.440
				132		^213^Po	4.2 μs	A	8
						^209^Tl	2.2 m	β^−^	0.659
				160		^209^Pb	3.5 h	β^−^	0.198
						^209^Bi	Stable	---	---
^223^Ra	11.4 d	α	5.7	108.4	50–70	^219^Ra	3.96 s	A	6.8
				126		^215^Po	1.78 ms	A	7.4
				140		^211^Pb	36.1 m	β^−^	---
						^211^Bi	2.13 m	A	6.6
								β^−^	---
						^211^Po	0.516 s	A	---
				128		^207^Tl	4.77 m	β^−^	---
						^207^Pb	Stable	---	---
^213^Bi	45.6 m	α	6	50–90	132	^213^Po	4.2 μs	A	8
		β^−^	0.444			^209^Tl	2.2 m	β^−^	0.659
		γ	0.440			^209^Pb	3.5 h	β^−^	0.198
						^209^Bi	Stable	---	---
^212^Bi	60.6 m	α	6.1	40–100	---	^212^Po	0.3 µs	A	---
		β^−^				^208^Tl	3.1 m	β^−^	---
						^208^Pb	Stable	---	---
^211^At	7.2 h	α	5.9	55–80	116	^207^Bi	33.4 y	β^−^	
		EC				^211^Po	0.516 s	A	---
						^207^Pb	Stable	---	---

**Table 2:** Structures of acyclic and macrocyclic common bifunctional reagents used with alpha-emitting radionuclides.

DOTA and Bifunctional Derivatives		Mainly Alpha Radionuclides	Ref
DOTA: 1,4,7,10-tetra-azacyclododecane-1,4,7,10-tetraacetic acid	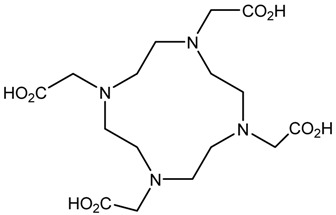		[32,36,37,38,39,40,41,42,43]
DOTAGA-anhydride	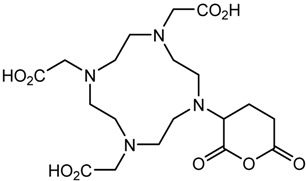	^212^Pb^2+^, ^225^Ac^3+^, ^212/213^Bi^3+^, ^223^Ra^2+^, ^149^Tb, ^226/227^Th	
DOTA-NHS-ester	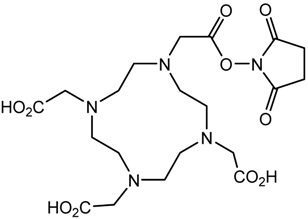		
DOTAGA, R = amide, DOTA-NHS-ester	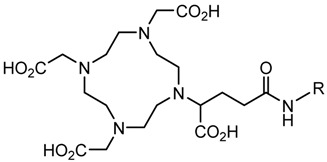		
*p*-SCN-Bn-DOTA (C-DOTA)	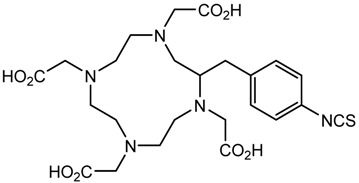		
DOTA derivatives TCMC, 3p-C-DEPA, and bifunctional derivatives			
3p-C-DEPA: 2-[(carboxymethyl)]-[5-(4-nitrophenyl-1-[4,7,10-tris-(carboxymethyl)- 1,4,7,10-tetraazacyclododecan-1-yl]pentan-2-yl)-amino]acetic acid	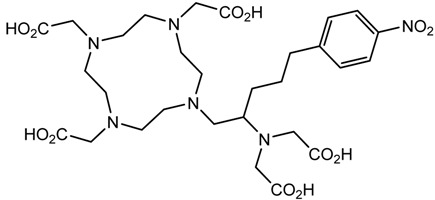		[32,44,45,46,47]
3p-C-DEPA-NCS	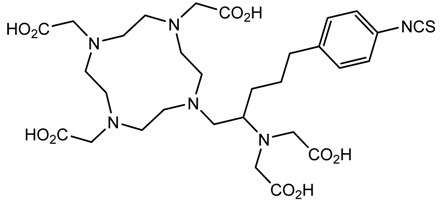		
TCMC, 1,4,7,10-tetrakis(carbamoylmethyl)- l,4,7,10-tetraazacyclododecane	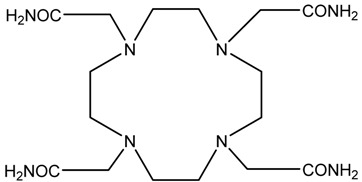	^212^Pb^2+^,^212/213^Bi^3+^	
*p*-SCN-Bn-TCMC	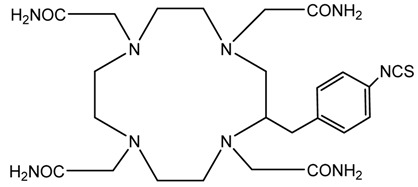		
NOTA, NETA, TACN-TM, and bifunctional derivatives			
NOTA: 1,4,7-triazacyclononane-1,4,7-triacetic acid	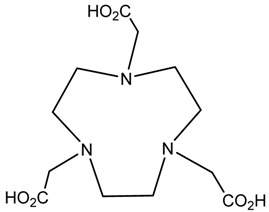		[32,43,48]
*p*-SCN-Bn-NOTA (C-NOTA)	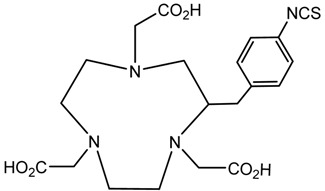		
NETA: {4-[2-(bis-carboxymethylamino)-ethyl]-7-carboxymethyl-[1,4,7]triazonan-1-yl}-acetic acid	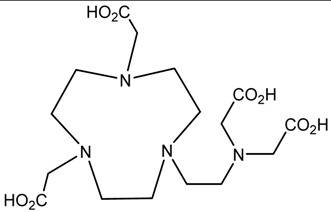		
C-NE3TA-NCS	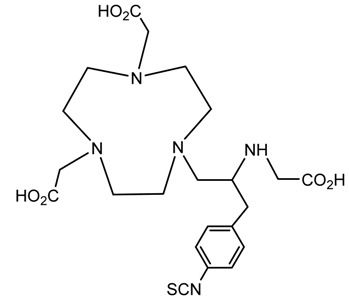	^212/213^Bi^3+^, ^212^Pb^2+^	
3p-C-NETA	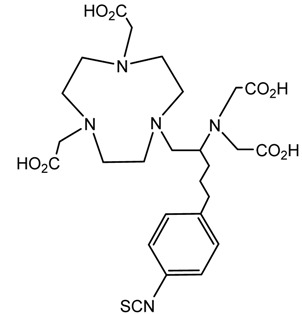		
C-NETA-NCS	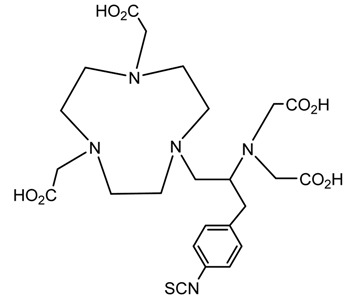		
DTPA, bifunctional derivatives, and others			
DTPA: diethylenetriaminepentaacetic acid	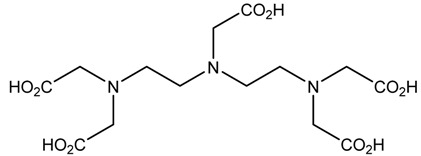		[5,32,36,41,42,49,50]
*p*-SCN-Bn-CHX-A″-DTPA	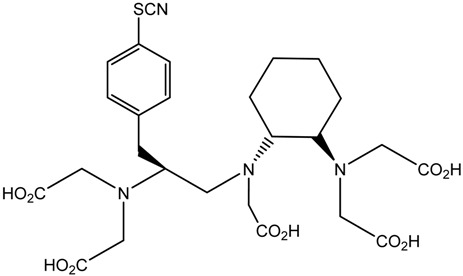		
*p*-SCN-Bn-1B-DTPA	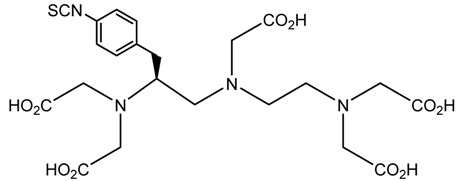		
CHX-A”-DTPA, 2-(p-isothiocyanatobenzyl)- cyclohexyldiethylenetriaminepentaacetic acid	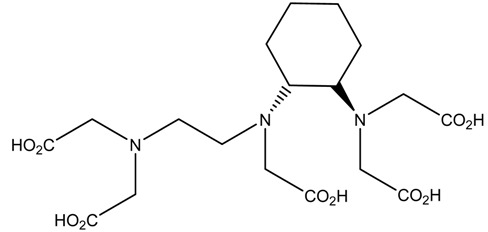	^212/213^Bi^3+^, ^225^Ac^3+^, ^223^Ra^2+^, ^149^Tb, ^226/227^Th	
*p*-SCN-Bn-1B4M-DTPA	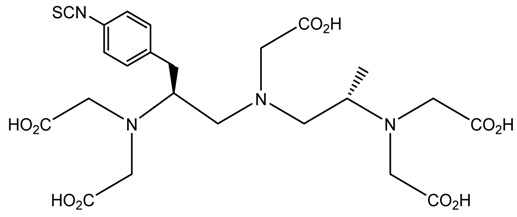		
(Me-2,3-HOPO)4-Bn-NCS	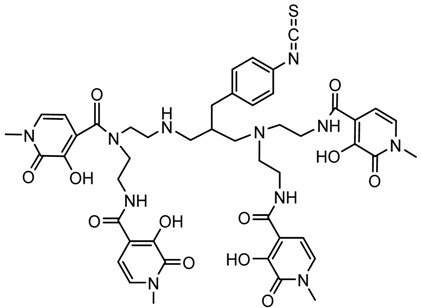	^226/227^Th	
HEHA, PEPA, and bifunctional derivatives			
HEHA: 1,4,7,10,13,16-hexaazacyclo-hexadecane-*N′,N″,N‴,N,N⁗,N″″′*-hexaacetic acid	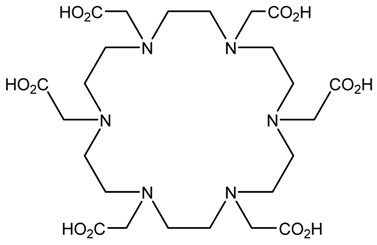		[32,36,37,38]
*p*-SCN-Bn-HEHA (C-HEHA)	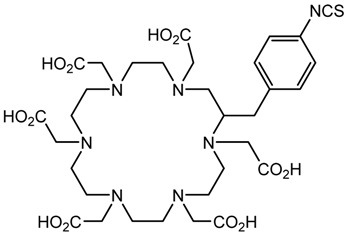		
PEPA: 1,4,7,10,13-pentaazacyclopentadecane-*N,N′,N″,N‴,N⁗*-pentaacetic acid	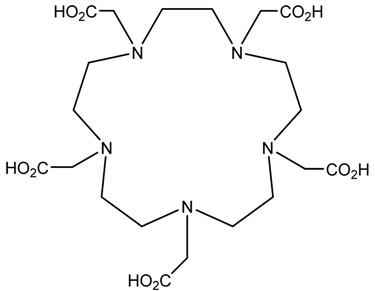		
*p*-SCN-Bn-PEPA (C-PEPA)	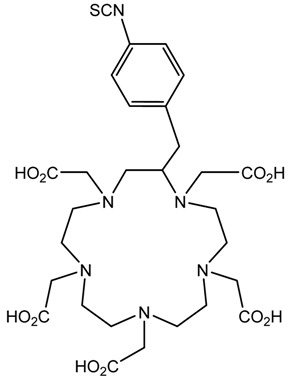		
MACROPA	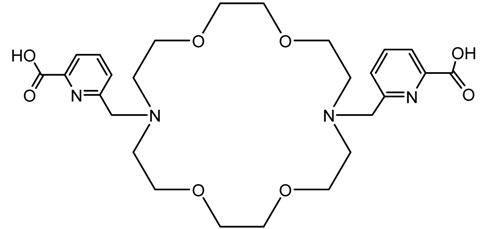	^225^Ac^3+^	

## Data Availability

Not applicable.
